# Reform of teaching and practice of the integrated teaching method BOPPPS-PBL in the course “clinical haematological test technique”

**DOI:** 10.1186/s12909-024-05765-9

**Published:** 2024-07-19

**Authors:** Xinrui Feng, Weiru Wu, Qinghua Bi

**Affiliations:** Department of Clinical Hematology, College of Pharmacy and Laboratory Medicine Science, Army Medical University, Chongqing, 400038 China

**Keywords:** Clinical hematology test technology, BOPPPS, PBL, Teaching reform

## Abstract

**Background:**

In order to meet the demand for laboratory talents in the clinical laboratory industry and address the current curriculum characteristics and shortcomings of the teaching mode of “Clinical Hematology Laboratory Technology”, we investigated the effectiveness of the bridge-in, objective, pre-assessment, participatory learning, post-assessment, and summary model combined with problem-based learning (BOPPPS-PBL) in undergraduate teaching of this course.

**Method:**

Seventy students majoring in Medical Laboratory Technology from the Army Medical University in the past 5 years have been selected and divided into two groups with the same teaching content and time. The control group (2015 and 2016 grades) used traditional teaching methods, while the experimental group (2017, 2018 and 2019 grades) used the BOPPPS-PBL model. After class, diverse evaluation methods were used to analyze the formative and summative exam scores of the two groups of students.

**Results:**

After the reform, students performed significantly better in exams than before. In addition, the new teaching methods have had a positive impact, with students demonstrating high motivation for self-directed learning and problem-solving abilities.

**Conclusion:**

Compared to traditional teaching methods. The BOPPPS-PBL integrated case study education model is a relatively effective teaching method to improve students’ problem-solving ability and comprehensive practical ability.

**Supplementary Information:**

The online version contains supplementary material available at 10.1186/s12909-024-05765-9.

## Background

Clinical hematology testing technology is a profound and comprehensive clinical discipline that emphasizes blood diseases as the research subject, rooted in theoretical knowledge of hematology, and employs physical, chemical, and immune testing experimental techniques as the tools [1]. This course is a pivotal course within laboratory medicine, primarily educating the morphology of blood cells in the form of tangible components in blood and bone marrow, prevalent red blood cell diseases, white blood cell diseases, hemorrhagic and thrombotic diseases, classification (typing), clinical manifestations, and laboratory examination methods [2]. Clinical hematology testing techniques emphasize the combination of theory and practice. It is a discipline closely integrated with clinical practice, which is helpful for the diagnosis, efficacy observation, and pre-monitoring of clinical hematological diseases [2].

With the advancement of China’s healthcare reform, the healthcare sector is transforming presently. In this milieu, the teaching plan and curriculum system of the main medical laboratory’s major also necessitate corresponding transformations, requiring students to uphold the fusion of “training objectives” and “employment needs”, focus on technical attributes, and actualize the amalgamation of educational training and clinical practical needs [3]. However, the conventional method of teaching presents several shortcomings. For instance, concurrent with the dynamic advancement of fundamental medical disciplines, testing methodologies and technologies have consistently innovated [4]. The diagnosis and management of blood diseases have undertaken substantial modifications, characterized by information, intelligence, and network integration [5, 6]. Novel clinical testing methods and instrumentation, such as flow cytometry, capillary electrophoresis, genetics and molecular biology, contribute significantly to the precise treatment, efficacy assessment, and prognosis insight into hematological disorders through uncomplicated, swift, and precise testing and analysis techniques [7, 8]. Nevertheless, the pertinent content within the instruction of traditional hematological testing techniques courses may be outdated or infrequently discussed. Meanwhile, experimental teaching is detached from clinical practice, and the procurement of some specialized specimens is challenging due to factors such as limited numbers of personnel and location, culminating in suboptimal teaching outcomes. The conventional method of education may no longer adequately address the training requirements of medical laboratory experts [9, 10]. Consequently, reform of the mode of education is imperative.

Innovative teaching methods can effectively improve the teaching quality of medical laboratory science and are an indispensable choice for improving clinical hematology laboratory technology courses [11]. The BOPPPS teaching model was suggested by the Canadian Instructional Skills Workshop (ISW) during the 1970s [12], which includes six stages: course introduction (B-Bridge in), learning objectives or outcomes (O-Objective or Outcome), pre assessment (P-Pre assessment), participatory learning (P-Participant Learning), post assessment (P-Post assessment), and summary (S-Summary). This model not only prioritizes student participation, but also improves the teaching ability and efficiency of teachers [13].

The Problem Based Learning (PBL) model, also known as Problem Based Teaching, originated in the 1950s. PBL has been described as an effective and efficient educational approach in the field of medical education [14, 15]. Medical courses aim to help students connect clinical knowledge with basic medical knowledge. However, traditional passive learning techniques are difficult to achieve. PBL focuses on problem-triggered and student-centered active learning strategies, which can improve students’ self-directed learning, interdisciplinary knowledge application, critical thinking, communication and collaboration abilities, as well as clinical thinking abilities [3, 16, 17]. Consequently, the harmonious fusion of case-based and PBL-based pedagogy, utilizing typical cases as teaching tools, assists students in comprehending and retaining the typical characteristics of diseases through reflection and analysis of typical cases, and also fosters student evidence-based reasoning [17].

The BOPPPS model has been applied to practical teaching in many disciplines, including biopharmaceutical engineering [18], thoracic surgery education [19], physiology education [20], and basic nursing education [21]. However, there are currently no reports on the application of BOPPPS and PBL models in clinical hematology laboratory technology teaching, both domestically and internationally. Although the BOPPPS and PBL models have been proven to be efficient and successful in improving students’ academic knowledge level, it is still unclear whether the BOPPPS-PBL model can play a good role in clinical hematology laboratory technology teaching in China. The integration of BOPPPS-PBL teaching technology aims to enhance students’ ability to analyze and solve problems, solving a crucial field in the learning and development of medical laboratory technology professionals.

## Methods

### Ethical approval

This study was performed in accordance with the Helsinki Declaration. The informed consent was obtained from all participants.

### Participants

The research is a non-randomized controlled trial. Due to the unique nature of military academies, the number of students admitted per session is relatively small. The participants comprised all 70 undergraduates from the Army Medical University who studied clinical hematology testing technology courses from August 2018 to December 2022 in four-year medical laboratory technology majors. All undergraduates studied clinical hematology testing techniques in the seventh semester. Nineteen undergraduates in 2015 and 2016 were included in the traditional teaching method group (control group), while 51 undergraduates in 2017, 2018, and 2019 were included in the BOPPPS-PBL group (BOPPPS-PBL group) (Table 3).

## Design

### Introducing clinical teaching guided by job competence

Our team has prioritized cultivating professional applied talents that are intimately connected with clinical practice in the realm of teaching design and reform. We utilized a collaborative clinical teaching model with clinical laboratories, enabling students to comprehend the application parameters and operational essentials of contemporary laboratory and diagnosis and treatment technology, and guiding them to further develop proficiency and mastery through practical operations. For instance, in the subject of hemorrhagic diseases and thrombotic diseases, in addition to imparting students with an understanding of the principles, methods, and significance of thrombus and hemostasis screening tests, it was also crucial to adeptly master modern testing techniques such as semi-automatic blood clotting instruments, thromboelastometers, and blood rheometers. Integrating advanced theory with industrial practice aims to foster medical laboratory technology professionals with core competitiveness in their roles.

### Teaching reform by introducing BOPPPS and case integration teaching model

In light of the unique attributes of the course “Clinical Hematology Testing Technology” and the practical teaching scenario, we have chosen to use the BOPPPS model for refinement and exploration, transforming the traditional cramming teaching mainly based on teacher lectures into student-centered active participatory learning [22, 23]. The incorporation of the BOPPPS model not only amplifies the cognitive capacity of students, invigorates their enthusiasm, but also enhances their understanding of abstract basic professional knowledge [24, 25].

**Fig. 1:**
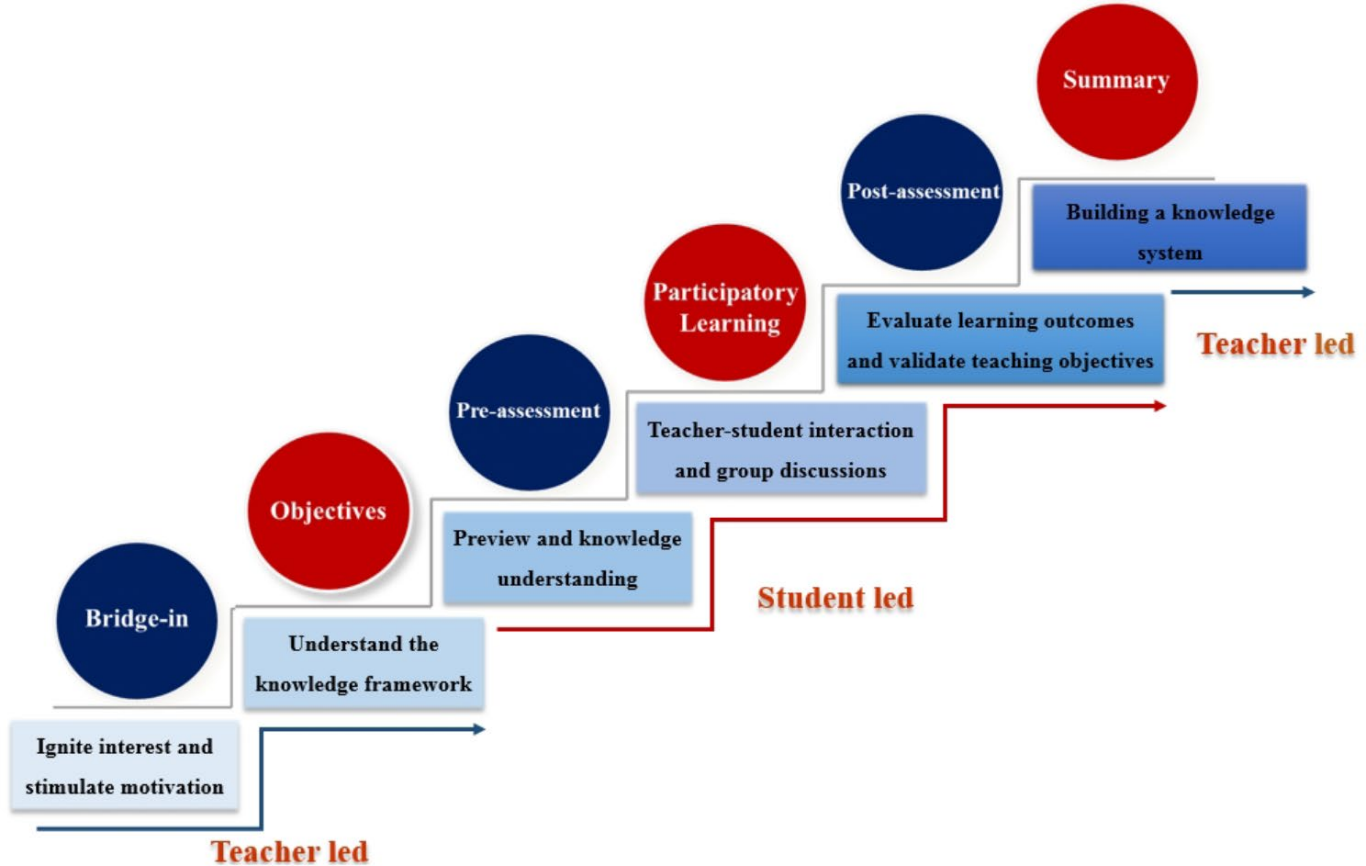
Design flowchart of BOPPPS model

According to the BOPPPS model, the teaching design and process of “iron deficiency anemia” in the course are divided into 6 modules (Fig. 1; Table 1) [26].

**Table 1:** Design of “iron deficiency anemia” based on BOPPPS model

BOPPPS	Teacher	Student
B- Bridge in	Bridge in: clinical cases Stimulating Thinking: What type of anemia? What is the cause of the disease?	Stimulate curiosity and actively participate in teaching
O-Objective or Outcome	Knowledge objective: To understand the etiology, clinical manifestations, and concepts of iron deficiency anemia; Master the diagnostic criteria, steps, and laboratory examination methods for iron deficiency anemia. Ability objective: Accurately identify blood and bone marrow cells of iron deficiency anemia, proficiently operate iron staining, analyze results, and issue bone marrow test reports. Quality objective: To highlight the cultivation of an overall conceptual system and logical thinking ability towards diseases.	Understand the knowledge framework of this lesson and remember the key and difficult points
P- Pre-assessment	Question 1: Definition of anemia Question 2: Classification of anemia Question 3: Precautions for diagnosing anemia	Participate in interaction, understand preview and knowledge understanding
P-Participatory Learning	1. Knowledge teaching: explaining what iron deficiency anemia is 2. Questioning and Thinking: The known causes of iron deficiency anemia 3. Inspiring thinking: How can laboratory tests be used to clarify the diagnosis of iron deficiency anemia? 4. Problem orientation: Analysis of typical cases, group discussion, selection of appropriate testing methods for operation 5. Discussion teaching: pre class arrangement, in class discussion, and post class writing of discussion summary report 6. Morphological identification: Ask about the identification of bone marrow and blood cell morphology 7. Experimental operation: Design a group experiment and issue a bone marrow test report	Constructing knowledge points based on “problems”, conducting operational discussions in groups, implementing each link, mobilizing students’ subjective initiative, and actively thinking and discussing
P- Post-assessment	Analyze each group’s mastery of student discussions, classroom performance, experimental operations, and bone marrow report forms	Grasp knowledge based on bone marrow cell morphology recognition, experimental operation process, and case analysis
S- Summary	Analyze and summarize the problems in teaching, and reflect and improve based on teaching objectives	Analyze and summarize the knowledge system and internalization situation

1. **Bridge-in (B)** Using a case-based teaching approach at the beginning of this lesson, the educational material is elucidated through clinical examples to kindle students’ curiosity, catalyzed learning enthusiasm, and quickly integrated students’ cognition into clinical scenarios.
2. **Objectives (O)** In the class, the teacher elaborated on the learning objectives and salient features of this lesson, and learners had an initial understanding of the theoretical framework of this module.
3. **Pre-assessment (P)** Pre-assessment was conducted to understand students’ mastery of overview knowledge and preview of new content, and subsequent teaching strategies were adjusted based on feedback data.
4. **Participatory Learning (P)** Participatory learning is the core of teaching activities. Through questioning, heuristic teaching, teacher-student interaction, and group discussions, the subjective initiative of students was fully mobilized, leading to positive thinking and discussion, and providing constructive feedback on their level of participation.
5. **Post-assessment (P)** Post-testing serves as an essential means of measuring student learning effectiveness and verifying curriculum objectives. Utilizing post-test outcomes, educators refined instructional methodology, while learners can understood their mastery of knowledge, thereby enhanced pedagogical effectiveness.
6. **Summary (S)** Finally, the teacher’s summary is a reflection on the teaching process, refining the course content, and further enhancing the instructional design. Through summarization, students constructed a knowledge framework, analyzed and summarized the internalization of knowledge, and enhanced learning effectiveness.

### Teaching reform by introducing PBL and case integration teaching model

Clinical hematology testing technology is a course closely related to clinical practice. In addition to emphasizing students’ mastery of professional theoretical knowledge of clinical hematological diseases and the principles of laboratory diagnostic methods, it also emphasizes the training of basic test skills, especially the knowledge of bone marrow cell morphology and the application of hematological disease test analysis in diagnosis, emphasizing the integration of theory with practice, Cultivate high-quality laboratory talents with dual abilities of “blood disease test skills” and “clinical blood disease diagnosis”. Therefore, having the ability to apply clinical practice is the main goal of this course teaching. To accomplish this, we focus on professional requirements, reflect on teaching methods, highlight teaching priorities, organically integrate problem-based (PBL) models with case-based teaching, and actively carry out teaching reforms.

Integrating PBL and case-based teaching in practical settings by developing a problem chain around real-world clinical hematological disease cases, with students as the main body and center of teaching activities, fully stimulating students’ thirst for knowledge. Case analysis seeks to align theory with practice, fostering students’ critical thinking, disease analysis, and problem-solving skills [27, 28].

Taking “Chronic Myelogenous Leukemia (CML)” as an example, this article introduces the design and implementation of PBL concept and case fusion teaching in this course. First, the true story of the protagonist prototype in the film “I’m Not the God of Medicine” is narrated to stimulate students’ interest in disease. Subsequently, using case studies as a guide, questions and guides students to think and solve problems from a clinical diagnosis and treatment perspective. For instance, what additional laboratory tests do you think are needed? What diseases do you consider possible based on current information? Through a series of questions, students are encouraged to follow the initial diagnosis of chronic myeloid leukemia patients, morphological characteristics of bone marrow imaging at different stages as the disease progresses, diagnostic techniques and key points, and outcome analysis and discussion, including the overall diagnostic process and strategic ideas for the final diagnosis. They are encouraged to understand the role of their knowledge in the diagnosis, treatment, and prognosis of hematological diseases in clinical practice, and to clarify their professional positioning, realizing the connection between theory and practice will enable students to have a more comprehensive and profound understanding of professional theoretical knowledge and testing techniques.

The PBL concept coupled with case fusion educational techniques strongly engages students in applying professional theoretical knowledge to practical clinical dilemmas, fostering their evidence-based thinking and problem-solving prowess [29, 30].

## Assessment methods and effectiveness evaluation

### Effectiveness assessment

Evaluation is the main way to evaluate students’ learning effectiveness, and it is also an important basis for testing the quality of teaching [31]. The traditional assessment method consists of two parts: theoretical assessment and experimental assessment, with a single and one-sided assessment method [32]. Students focus only on the final assessment, resulting in low participation in the teaching process, resulting in low learning ability and overall quality.

Based on the characteristics of the course “Clinical Hematology Testing Technology”, a diversified assessment system combining formative assessment and summative assessment is adopted. After the reform of assessment methods, formative assessment is a diversified evaluation method that emphasizes the organic combination of assessment process and assessment indicators. The diversified assessment process can flexibly adopt different assessment methods based on students’ personalities and needs, continuously improve in the process, achieve the full process and integration of course assessment, and leverage the assessment, guidance, education, and encouragement functions of evaluation methods [33, 34]. The diverse assessment indicators encompass three primary components: homework, experimental operation, and final exam. The aim is to avoid the drawbacks of the traditional single assessment form of “examination instead of evaluation”, and to prevent student contingency and one-sided performance.

**Fig. 2:**
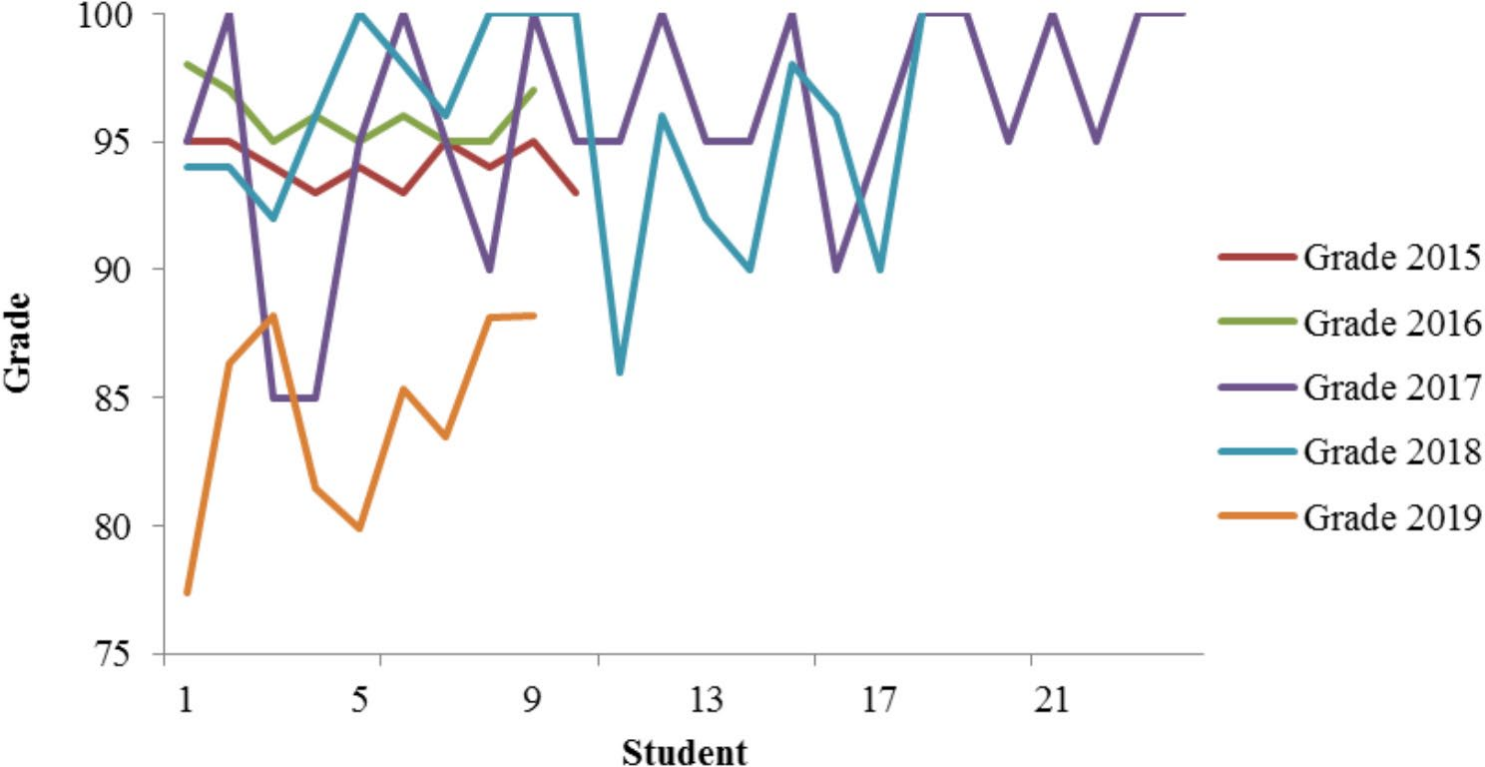
Comparison of students’ daily scores and formative assessment scores BOPPPS-PBL group and control group

This course adopts a comprehensive, multi-level and multi-dimensional assessment method by considering the teaching hours and content composition comprehensively. The usual assignments in formative assessment are designed around the learning objectives of the course, such as: assessment of students’ understanding of the morphology of peripheral blood and bone marrow cells, self-made forms and flow charts inductive reasoning of basic theoretical knowledge of various diseases, and knowledge internalization understanding; The experimental operations include self-designed experiments, bone marrow cell morphology recognition and case analysis, bone marrow smear analysis and diagnosis, providing case information and supporting bone marrow smear, completing the reading and writing of bone marrow test reports, including diagnostic opinions and differential diagnosis, mainly examining students’ mastery of basic methods and skills such as morphological recognition, and judging their clinical diagnostic thinking and other abilities based on case analysis, diagnosis, and other content; The summative assessment mainly assesses students’ mastery of basic theoretical knowledge, laboratory examinations, and other basic methods and skills. From the perspective of clinical diagnosis and treatment, it examines students’ choice of test methods, diagnostic logic, and result analysis, breaking the tradition of memorization, emphasizing understanding over memory, requiring students to grasp basic concepts and operating procedures, and proficiently using specific methods to solve diagnostic problems related to clinical blood diseases and other related cases. Before and after the reform, the daily performance is shown in Table 3 and Fig. 2. The evaluation method for daily performance prior to the reform was subjective and limited, with a low degree of differentiation. After the reform, the adoption of a diversified formative assessment method significantly improved the differentiation of grades. In the standardized formative assessment, students’ overall performance in the curriculum is assessed more objectively and comprehensively (Table 2).

**Table 2:** Assessment methods of clinical hematology test technology

Assessment composition	Grades composition	Percent	Assessment form	Evaluation points
Formative assessment	Assignments	10	Participatory learning	1. Advance distribution of materials 2. Discussion, group speaking 3. Write a seminar summary report
			Classroom test	1. Morphological identification of peripheral blood and bone marrow cells 2. In-house questioning
			Experimental report writing	1. Diagnostic opinions 2. Differential diagnosis 3. Bone marrow examination report 4. Experimental analysis report form
			Homework after class	1. Make tables and flow charts inductive reasoning 2. Determination of peripheral blood and bone marrow cell morphology 3. Case analysis and diagnosis
	Experimental Operations	30	Group collaboration	1. Design experiments, team collaboration 2. Typical case analysis, group discussion, and selection of appropriate testing methods for operation
			Experimental Operations	1. Cytochemical staining, POX staining, glycogen staining, and esterase staining, etc. 2. Reading morphological films 3. Standardization of experimental operation 4. Rationality of self-designed experiments
			Problem analysis and solving	Analysis and diagnosis of typical cases, group discussion, and selection of appropriate testing methods for operation
Final assessment	End of class exam	60	Closed book written test	The comprehension question type is greater than the memory question type

### Statistical analysis

All statistical analyses were carried out using SPSS 22.0 (SPSS, Inc., Chicago, IL). Measurement data are expressed as the means ± SD and analyzed by t-test. Categorical data were analyzed by the chi-square test. Statistical significance was defined as *p* < 0.05.

## Results

The general characteristics of the two groups are shown in Table 3. The four-year undergraduate medical laboratory technology students in 2015 and 2016 (the control group) comprised 19 individuals, including 4 males and 15 females. The mean age of the control group was 20.25 years. The four-year Medical Laboratory Technology undergraduate group in 2016, 2017 and 2018 (the BOPPPS-PBL group) comprised 51 individuals, 34 of whom were male and 17 were female. The mean age of the BOPPPS-PBL group was 20.32 years. No significant differences in general characteristics, including sex and age, were found between the two groups (*p* > 0.05).

**Table 3:** Characteristics of the participants of the two groups

Characteristics	Control group (*n* = 19)	BOPPPS-PBL group(*n* = 51)	t/X^2^	*p*
Age (years), (mean ± SD)	20.32 ± 0.09	20.25 ± 0.15	1.39	0.24
Sex			1.02	0.16
Male, n (%)	15(78.9)	34(66.7)		
Female, n (%)	4(21.1)	17(33.3)		

The formative and standardized assessment and evaluation methods are shown in Table 2. The results before and after the formative assessment score reform are shown in Table 4; Fig. 2. The control group’s evaluation method for daily performance was subjective and limited, with a low degree of differentiation. There was no statistically significant difference in formative evaluation scores between grades 2015 and 2016 (*p* > 0.05). Yet, the BOPPPS-PBL group’s subsequent adoption of diverse formative assessment methods, and the difference in formative assessment scores between the three groups from 2017 to 2019 was statistically significant (*p* < 0.05), which significantly improved their grade discrimination. This shift towards standardized formative assessment provided an objective and comprehensive account of student performance.

**Table 4:** Comparison of students’ formative assessment scores BOPPPS-PBL group and Control group

Group	Grade	Number	Max	Min	Average	Standard deviation
Control	2015	10	95	93	94.10	0.83
	2016	9	98	95	96.00	1.05
BOPPPS-PBL	2017	24	100	85	95.83	4.50
	2018	18	100	86	95.44	4.04
	2019	9	88	77	84.26	3.74

**Fig. 3:**
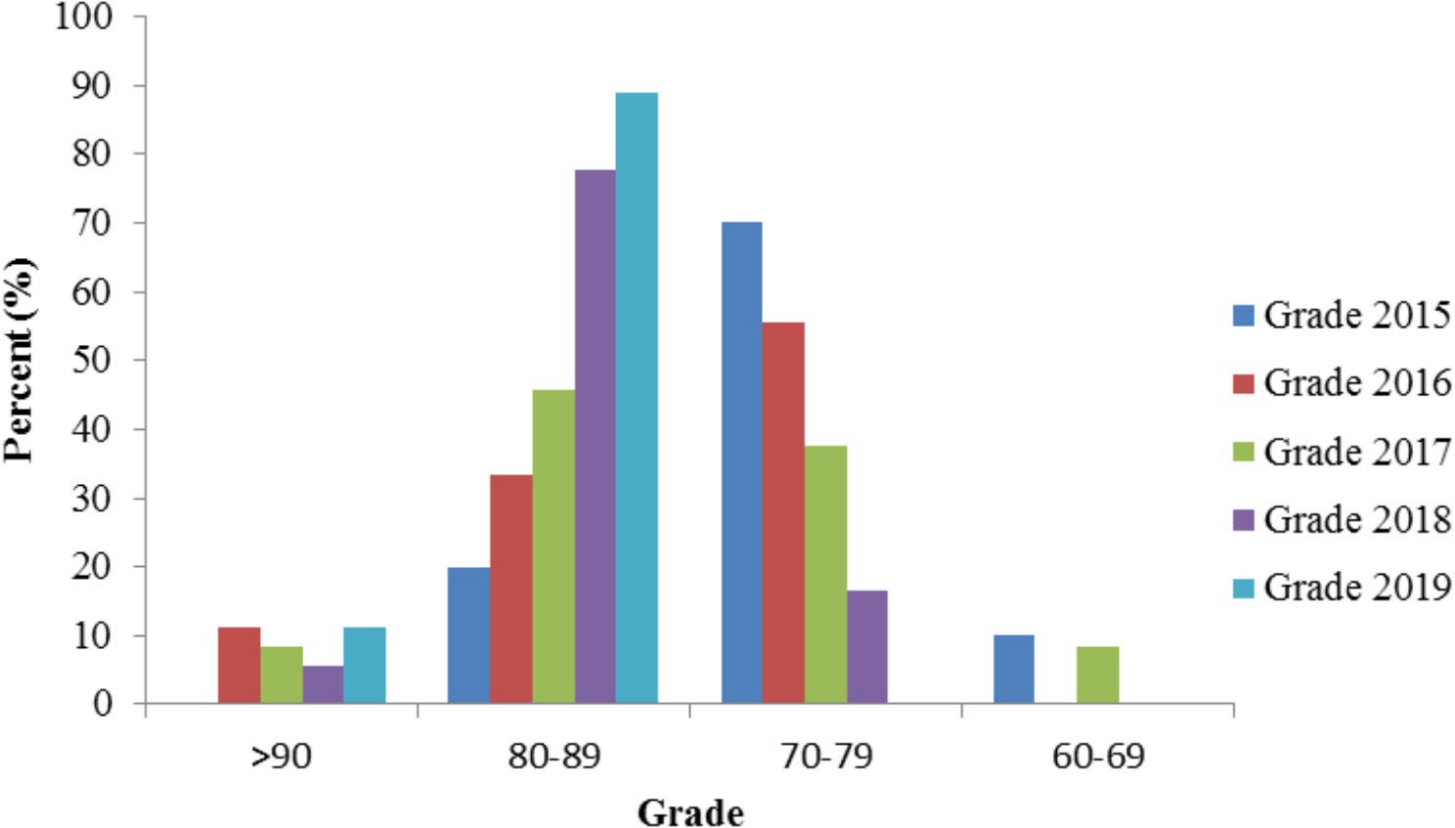
Comparison of score ranges for students’ final assessment BOPPPS-PBL group and control group

**Fig. 4:**
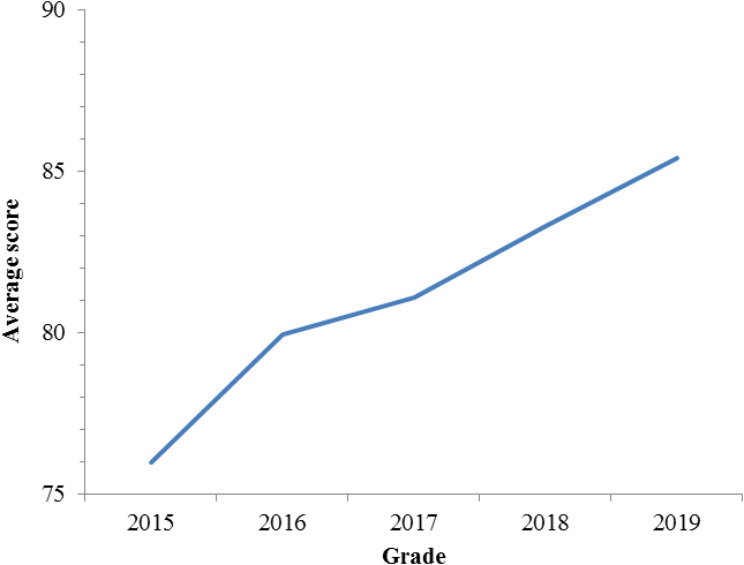
Comparison of average scores of students in final assessment BOPPPS-PBL group and control group

Post-teaching reform, the BOPPPS-PBL group achieved higher summary evaluation scores than the control group (*p* < 0.01), with a statistically significant difference (*p* < 0.05). Additionally, the proportion of high scoring students in the final evaluation significantly increased (Fig. 3), and the difference was statistically significant (*p* < 0.05) (Fig. 4).Evidently, the integration of BOPPPS-PBL teaching philosophy and case-based methodologies has yielded positive outcomes among these students. However, this study does have certain constraints. As a minor major in medical laboratory technology, the student sample size is insufficient, comprising only 70 students.

## Discussion

Clinical hematology testing technology is a course with professional characteristics. In recent years, medical education has shifted from teacher-centered to learner-centered, and the apprenticeship model has shifted to a new type of education model that focuses on skill development [35]. Medical laboratory technology belongs to niche majors, where students mainly learn basic theoretical knowledge in basic medicine and medical laboratory, and receive systematic training in medical laboratory operation skills [36]. Many innovative educational models have been developed both domestically and internationally in terms of course content, teaching design, and teaching methods related to medical laboratory technology, in order to strengthen the teaching of medical laboratory technology [37, 38]. However, in the process of teaching mode transformation, there are still many problems in medical laboratory technology education.

In China, clinical hematology testing technology is a branch of laboratory medicine. This course is a professional course in the four-year undergraduate medical laboratory technology teaching of the Department of Pharmacy and Laboratory Medicine at the Army Medical University. Traditional teaching methods mainly have the following characteristics: (1) As students need to work in clinical laboratory positions in the future, limited experimental teaching can only provide students with a preliminary understanding of normal and pathological bone marrow and blood morphology, making it difficult to lay a solid foundation for future work. In addition, the experimental teaching content is limited by outdated instruments and equipment, and the understanding of new and high-throughput testing methods and technologies in clinical practice is limited to the basic theoretical level, making it difficult to cultivate high-quality talents who are closely connected with clinical practice. (2) Current teaching methodologies are limited and heuristic instruction is insufficient. Typically, teachers first teach theoretical knowledge and then explain experimental operations. This cramming teaching methodology limits students’ subjectivity, contributes to subpar participation and diminished motivation [39]. Concurrently, this passive study approach often prevents students from fully comprehending experimental principles and operations, and from effectively applying fundamental theoretical knowledge to practical applications [40]. (3) It is difficult to comprehensively evaluate students’ learning outcomes through assessment methods that focus primarily on theoretical assessment and experimental assessment results. Therefore, traditional teaching methods can no longer meet the needs of medical students, and new teaching methods are constantly being developed and improved by educators.

This study applies the BOPPPS-PBL model to the teaching of clinical hematological testing techniques. Compared with traditional teaching methods, the BOPPPS-PBL model, based on the two single teaching modes of BOPPPS and PBL, has the following advantages. Firstly, based on basic professional knowledge, closely adhering to professional needs, combined with the characteristics of the laboratory profession, highlighting the combination of “theory testing disease”. Based on typical clinical cases, stimulate students’ interest, improve learning effectiveness, and reinforce students’ mastery of basic theories and comprehensive practical abilities in this discipline [41].

Secondly, the BOPPPS-PBL model has changed the traditional teaching relationship. Classroom teaching is student-centered, and more emphasis is placed on teacher-student interaction. In participatory learning, students purposefully acquire knowledge and combine theory with practice through analysis of practical cases. [42].

Finally, this study carefully examined quantitative data and found that the BOPPPS-PBL group had significantly higher summary evaluation scores than the control group (Table 5). Meanwhile, the comparison of the score range between the BOPPPS-PBL group and the control group in the summative assessment showed a significant increase in the proportion of BOPPPS-PBL in the high score range of 80–89, and the 2019 grade students were in the good and excellent score range (Fig. 3), proving that this method significantly improved students’ understanding and practical ability of theoretical principles. In addition, compared with the control group, the average grades of students using the BOPPPS-PBL method also improved significantly, which proves the effectiveness of the BOPPPS-PBL method. Firstly, the BOPPPS model is based on constructivist and humanistic learning theories [43], while PBL is mainly based on constructivist cognitive theory and social constructivist [44]. BOPPPS-PBL, as an innovative medical teaching method, promotes students to transform from passive external stimulus recipients and indoctrination recipients into active information processors and meaning constructors. This transformation process encourages mastery, internalization, and absorption of knowledge [45]. Furthermore, in the BOPPPS-PBL model, BOPPPS divides the learning process into multiple modules, each of which can attract and motivate students. Simultaneously combine the benefits of the PBL learning process based on real cases. Our research findings indicate that the BOPPPS-PBL model promotes the cultivation of clinical practice skills and the learning of core competencies, ultimately benefiting students.

**Table 5:** Comparison of students’ summative assessment scores BOPPPS-PBL group and control group

Group	Grade	Number	Max	Min	Average	Standard deviation
Control	2015	10	89.00	65.50	76.00	6.67
	2016	9	91.75	73.50	79.96	6.08
BOPPPS-PBL	2017	24	96.00	59.50	81.08	7.78
	2018	18	93.54	75.18	83.30	4.36
	2019	9	92.26	81.16	85.41	3.46

### Limitations

This study evaluated the effectiveness of comparing the control group and the BOPPPS-PBL group through quantitative research design. The results of this study demonstrate that it was successful and beneficial. Nevertheless, the study presents certain constraints. Firstly, the sample size was small. Research with additional samples are needed to validate the effect of the BOPPPS-PBL model. Secondly, the comparison between BOPPPS and PBL models was not distinctively examined in this study. This deficiency can be rectified in subsequent research. Thirdly, this study focused exclusively on the effect within one course, future validation studies could encompass diverse courses.

## Conclusions

In the context of upgrading precision medicine to a “national strategy”, medical lab technology – the “eye of medicine” - experiences extensive advancement. Clinical hematology laboratory technology has become an indispensable and important means for clinical diagnosis, treatment, and prognosis judgment of blood diseases. Therefore, it is necessary to systematically teach hematological testing knowledge to undergraduate medical laboratory students.

In summary, by combining the integrated teaching concept of BOPPPS-PBL with case-based teaching methods in the exploration and practice of teaching reform of “clinical hematology test technology”, and expanding the evaluation system, the comprehensive evaluation of students is carried out from multiple dimensions of “knowledge, ability, and quality”. In the practice of education and teaching, the author’s team has achieved some gains and results, achieving students’ dominant position in talent cultivation, combining the academic nature of knowledge with the fun of learning, and enhancing students’ ability to learn independently, think, analyze, and solve problems. However, in the teaching summary, it was found that it is necessary to adapt flexibly to teaching activities based on student formative assessment and experimental performance, and further improve it, in order to cultivate high-quality medical laboratory professionals who can connect with clinical practice and application.

### Electronic supplementary material

Below is the link to the electronic supplementary material.

Supplementary Material 1

Supplementary Material 2

Supplementary Material 3

Supplementary Material 4

## Data Availability

Data is provided within the related files.
