# Supplementary figures and images for: Reform of teaching and practice of the integrated teaching method BOPPPS-PBL in the course “clinical haematological test technique”
Source: BMC Med Educ. 2024 Jul 19;24:773. doi: 10.1186/s12909-024-05765-9 (PMC11264887; doi:10.1186/s12909-024-05765-9)

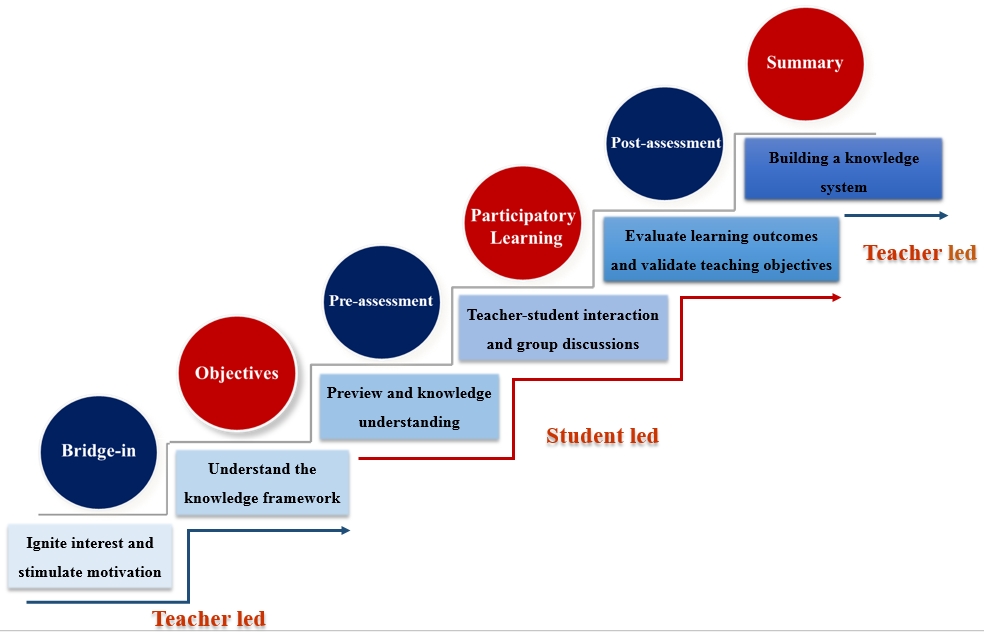

Supplement: Supplementary file 1 — Supplementary Material 1 [file 12909_2024_5765_MOESM1_ESM.png]

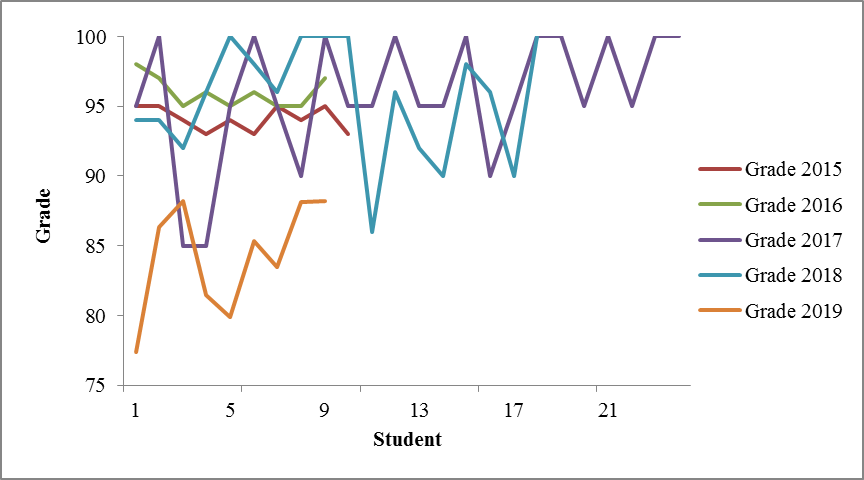

Supplement: Supplementary file 2 — Supplementary Material 2 [file 12909_2024_5765_MOESM2_ESM.png]

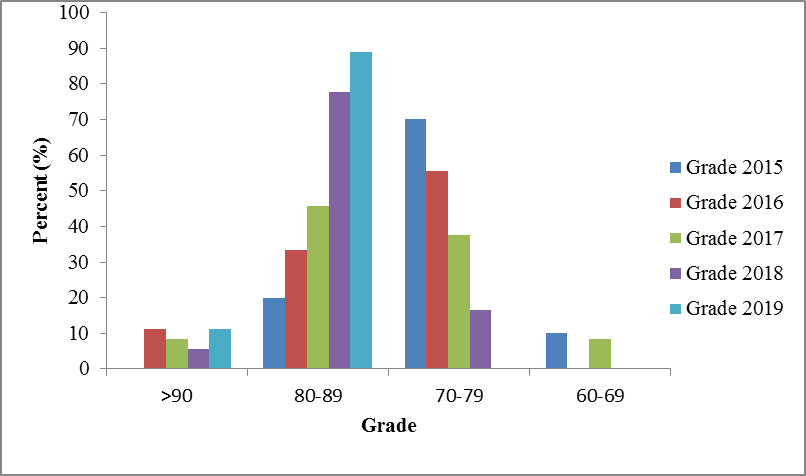

Supplement: Supplementary file 3 — Supplementary Material 3 [file 12909_2024_5765_MOESM3_ESM.png]

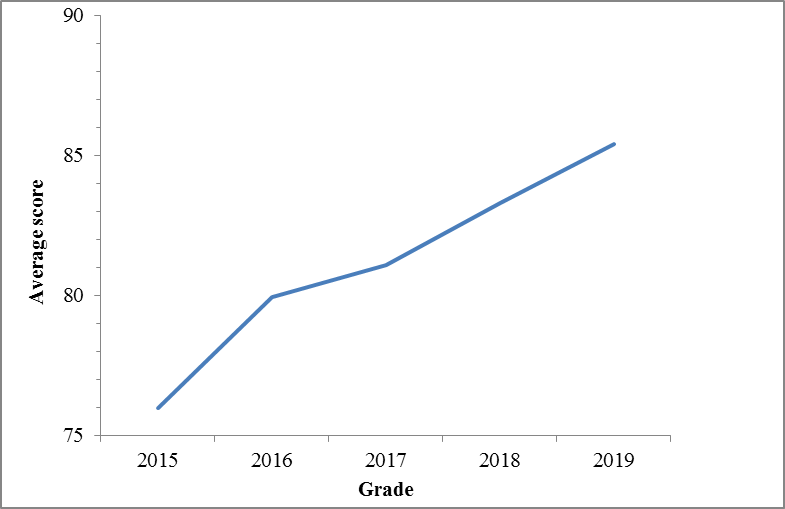

Supplement: Supplementary file 4 — Supplementary Material 4 [file 12909_2024_5765_MOESM4_ESM.png]
